# The Effectiveness of Albumin-to-Globulin Ratio as a Prognostic Biomarker in Primary Gastrointestinal Cancer: A Systematic Review and Meta-Analysis

**DOI:** 10.7759/cureus.61830

**Published:** 2024-06-06

**Authors:** William Delladio, Crystal Barroca, Will S Roberts, Hoang Nguyen

**Affiliations:** 1 Osteopathic Medicine, Nova Southeastern University Dr. Kiran C. Patel College of Osteopathic Medicine, Clearwater, USA; 2 Basic Sciences, Nova Southeastern University Dr. Kiran C. Patel College of Osteopathic Medicine, Clearwater, USA

**Keywords:** gastrointestinal cancer, esophageal cancer, gastric cancer (gc), colorectal cancer, cancer prognosis, albumin-to-globulin ratio, serum albumin, globulin

## Abstract

Albumin-to-globulin ratio (AGR) is a cheap, widely accessible component of common blood work that has been implicated in the prognosis of various cancers. This effect is attributed to the cooperative relationship between albumin reflecting the body’s nutritional status and globulin serving as an indicator of immune status. With the high morbidity and mortality associated with gastrointestinal cancer and the increasing necessity for cost-effective health care, research into AGR’s potential as an indicator of prognosis is warranted. A database search, including key terms between AGR and gastrointestinal cancer, was performed. Random-effects meta-analysis was completed on extracted hazard ratios with two-sided p-values <0.05 being deemed significant. A total of 8,384 patients with gastrointestinal cancer were included. A low AGR was found to be associated with increased risk for reduced overall survival in cancer of the primary GI tract (HR: 1.82, 1.35-2.45, p < 0.001), esophageal cancer (HR: 1.57, 1.19-2.06, p < 0.001), colon cancer (HR: 3.36, 2.02-5.58, p < 0.001), and colorectal cancer (HR: 2.27, 1.15-4.48, p = 0.02) populations. A low AGR is significantly associated with increased risk for reduced overall survival in primary gastrointestinal cancer. Due to the ease of access and low cost to physicians and patients, incorporation of AGR into clinical evaluation of prognosis in these cancers should prove beneficial to patient outcomes.

## Introduction and background

Although the overall death rate of cancer has declined by 33% since 1991, cancer remains the second leading cause of death in the United States, second only to heart disease [1]. On a global scale, gastrointestinal (GI) cancer specifically poses a significant threat to public health as it accounts for 26% of all cancers, and 35% of all cancer-related deaths [2]. Despite the continued growth and advancements in the detection and treatment of these malignancies, the expected new cases of GI cancer diagnoses and deaths are expected to increase by 58% and 78%, respectively, by 2040. [2] This necessitates continued effort and research into combating this global threat in hopes of improving the health outcomes for those suffering from a cancer diagnosis involving the GI tract.

A recent study of patients in Japan found the diagnosis of stage III and IV GI malignancies has increased since the COVID-19 pandemic. In contrast, stages I and II have declined, likely due to delays in routine screening practices given the nature of the pandemic allowing for the progression of undiagnosed malignancies [3]. The nature of many of these malignancies allows for curative resection; however, the mortality remains high. The same study indicated decreased overall survival for individuals with colon cancer and rectal cancer when treatment was delayed [3].

Early intervention and efficient decision-making in management strategies is imperative in these patients. The use of albumin-to-globulin ratio (AGR) is gaining traction as an efficient, cost-effective, and readily accessible means of predicting the prognosis of many malignancies. Serum albumin is an indicator of nutritional status and globulin has been shown to be involved in immune processes and inflammatory response [4,5]. Albumin is a dynamic protein in the human body with a wide array of functions. Primarily, albumin regulates fluid distribution in the body as it is the main component of osmotic pressure, allowing it to stabilize the balance between intracellular and extracellular fluids [6,7]. Secondarily, albumin plays a role in neutralizing toxins, binding inflammatory mediators and immune response receptors, and extracellular transportation of many compounds [8]. Historically, albumin has been used to approximate nutritional status as synthesis in the liver is regulated by feedback of osmotic pressure, inflammatory state, and nutrition [9]. Globulin is a broad term used to describe a wide variety of serum proteins, including immunoglobulins, acute phase reactants, carrier proteins, and complement factors [9]. Historically, globulin has been used to approximate the inflammatory status of the human body, as serum globulins are rapidly elevated in the setting of inflammation [10].

Both albumin and globulin provide valuable insight into a patient's current overall health and immune status as they begin the process of fighting their cancer diagnosis. Poor nutrition contributes to decreased chemotherapeutic efficacy and inflammation at the site of tumor microenvironment contributes to the progression of malignancy. The synergistic use of these two serum blood markers in the AGR offers physicians a valuable tool in streamlining the treatment decision-making process as it guides them in determining how aggressive of an approach to implement with the initial management of these cancers [11].

## Review

Materials & methods

A database search was performed in Embase, Web of Science, and MEDLINE-PubMed searching for all articles that included keywords relating any form of prognosis of cancer of the primary gastrointestinal (GI) tract to albumin-to-globulin ratio (AGR). The exact search string is reported in Table 1.

**Table 1:** Database search string. This table depicts the precise search string utilized to extract the initial relevant literature from Embase, MEDLINE-PubMed, and Web of Science databases.

Embase - 203 results	
1	‘albumin*’:ti,ab,kw
2	‘globulin*’:ti,ab,kw
3	‘prognosis*’:ti,ab,kw OR ‘survival*’:ti,ab,kw OR ‘mortality*’:ti,ab,kw OR ‘lifespan*’:ti,ab,kw OR ‘recurrence*’:ti,ab,kw
4	‘cancer*’:ti,ab,kw OR ‘neoplasm*’:ti,ab,kw OR ‘tumor*’:ti,ab,kw OR ‘metastasis*’:ti,ab,kw OR ‘malignancy*’:ti,ab,kw OR ‘carcinoma*’:ti,ab,kw OR ‘lymphoma*’:ti,ab,kw OR ‘sarcoma*’:ti,ab,kw
5	‘gastrointestinal*’:ti,ab,kw OR ‘GI*’:ti,ab,kw OR ‘oral*’:ti,ab,kw OR ‘mouth*’:ti,ab,kw OR 'oropharynx*’:ti,ab,kw OR ‘oropharyngeal*’:ti,ab,kw OR ‘esophagus*’:ti,ab,kw OR ‘esophageal*’:ti,ab,kw OR ‘stomach*’:ti,ab,kw OR ‘gastric*’:ti,ab,kw OR ‘bowel*’:ti,ab,kw OR ‘intestine*’:ti,ab,kw OR ‘intestinal*’:ti,ab,kw OR ‘duodenum*’:ti,ab,kw OR ‘jejunum*’:ti,ab,kw OR ‘ileum*’:ti,ab,kw OR ‘colon*’:ti,ab,kw OR ‘rectum*’:ti,ab,kw OR ‘rectal*’:ti,ab,kw OR ‘anus*’:ti,ab,kw OR ‘anal*’:ti,ab,kw
6	#1 AND #2 AND #3
7	#4 AND #5
8	#6 AND #7
9	#8 AND (2013:py OR 2014:py OR 2015:py OR 2016:py OR 2017:py OR 2018:py OR 2019:py OR 2020:py OR 2021:py OR 2022:py OR 2023:py) AND 'article'/it
MEDLINE - PubMed - 167 results	
1	albumin[Title/Abstract] or albumin[MeSH Terms]
2	globulin[Title/Abstract] OR globulin[MeSH Terms]
3	prognosis[Title/Abstract] OR prognosis[MeSH Terms] OR survival[Title/Abstract] OR survival[MeSH Terms] OR mortality[Title/Abstract] OR mortality[MeSH Terms] OR lifespan[Title/Abstract] OR lifespan[MeSH Terms] OR recurrence[Title/Abstract] OR recurrence[MeSH Terms]
4	cancer[Title/Abstract] OR cancer[MeSH Terms] OR neoplasm[Title/Abstract] OR neoplasm[MeSH Terms] OR tumor[Title/Abstract] OR tumor[MeSH Terms] OR metastasis[Title/Abstract] OR metastasis[MeSH Terms] OR malignancy[Title/Abstract] OR malignancy[MeSH Terms] OR carcinoma[Title/Abstract] OR carcinoma[MeSH Terms] OR lymphoma[Title/Abstract] OR lymphoma[MeSH Terms] OR sarcoma[Title/Abstract] OR sarcoma[MeSH Terms]
5	gastrointestinal[Title/Abstract] OR gastrointestinal[MeSH Terms] OR GI[Title/Abstract] OR GI[MeSH Terms] OR oral[Title/Abstract] OR oral[MeSH Terms] OR oropharynx[Title/Abstract] OR oropharynx[MeSH Terms] OR oropharyngeal[Title/Abstract] OR oropharyngeal[MeSH Terms] OR esophagus[Title/Abstract] OR esophagus[MeSH Terms] OR esophageal[Title/Abstract] OR esophageal[MeSH Terms] OR stomach[Title/Abstract] OR stomach[MeSH Terms] OR gastric[Title/Abstract] OR gastric[MeSH Terms] OR bowel[Title/Abstract] OR bowel[MeSH Terms] OR intestine[Title/Abstract] OR intestine[MeSH Terms] OR duodenum[Title/Abstract] OR duodenum[MeSH Terms] OR jejunum[Title/Abstract] OR jejunum[MeSH Terms] OR ileum[Title/Abstract] OR ileum[MeSH Terms] OR colon[Title/Abstract] OR colon[MeSH Terms] OR rectum[Title/Abstract] OR rectum[MeSH Terms] OR rectal[Title/Abstract] OR rectal[MeSH Terms] OR anus[Title/Abstract] OR anus[MeSH Terms] OR anal[Title/Abstract] OR anal[MeSH Terms]
6	#1 AND #2 AND #3
7	#4 AND #5
8	#6 AND #7
9	(#8) Full-Text AND PY(2013 - 2023)
Web of Science - 78 results	
1	TS=(albumin)
2	TS=(globulin)
3	TS=(prognosis OR survival OR mortality OR lifespan OR recurrence)
4	TS=(cancer OR neoplasm OR tumor OR metastasis OR malignancy OR carcinoma OR lymphoma OR sarcoma)
5	TS=(gastrointestinal OR GI OR oral OR oropharynx OR oropharyngeal OR esophagus OR esophageal OR stomach OR gastric OR bowel OR intestine OR duodenum OR jejunum OR ileum OR colon OR rectum OR rectal OR anus OR anal)
6	#1 AND #2 AND #3
7	#4 AND #5
8	#6 AND #7
9	(#8) Article AND PY(2013-2023)

**Figure 1:**
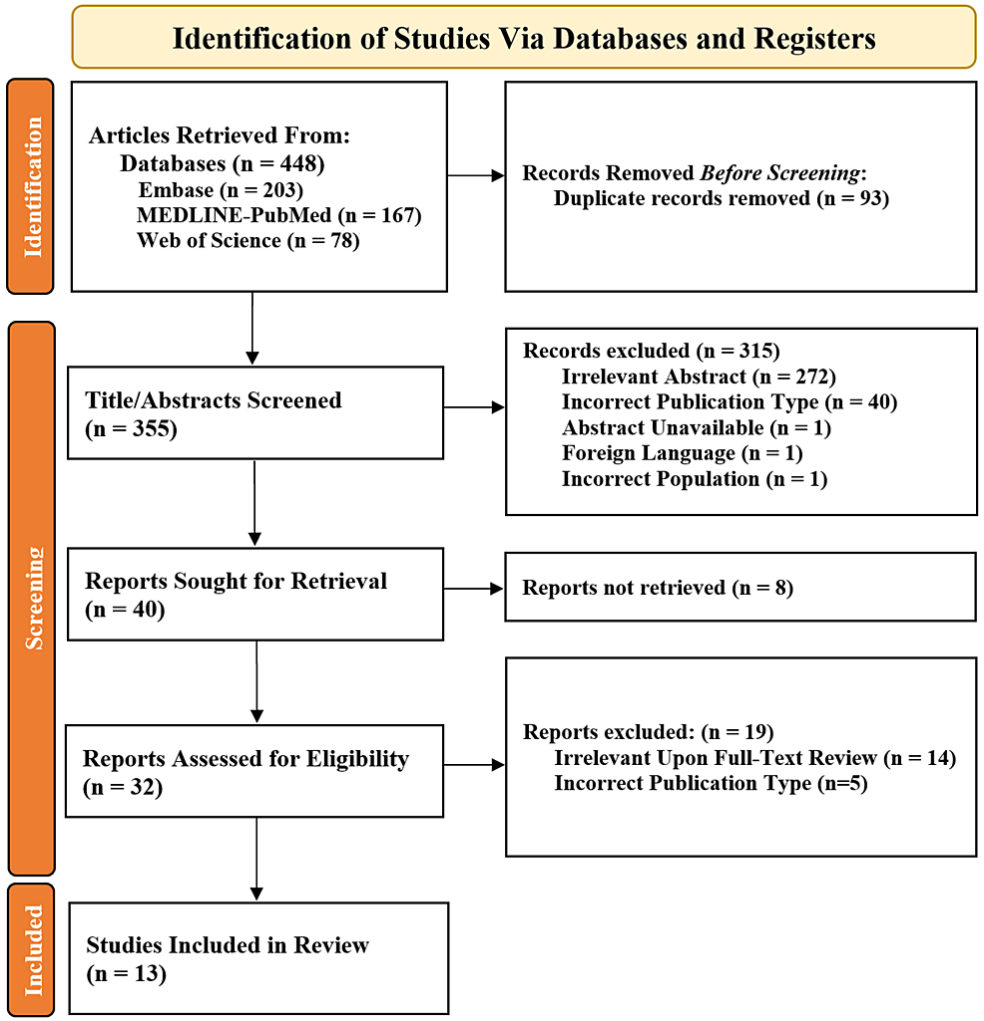
Systematic PRISMA diagram. This figure depicts the PRISMA diagram summarizing the article search, screening, and inclusions. PRISMA: Preferred Reporting Items for Systematic Reviews and Meta-Analyses.

Further filters were applied to search for studies from 2013 to 2023 and only studies with an associated full-text. In total, 448 records were identified, which were subsequently deduplicated to 355 unique results (Figure 1). The 355 unique articles were imported into a blinded article screening software titled Rayyan (Rayyan Systems Inc., Cambridge, MA) and screened by the first two authors [12]. Standardized inclusion/exclusion criteria were applied to the abstracts. Specifically, abstracts were excluded if they were in (1) a foreign language, (2) the wrong publication type, (3) a non-adult human study, and (4) not relevant. Application of the aforementioned criteria to the 355 abstracts yielded 40 studies that met all inclusion standards. A second blinded screen was performed in Rayyan on the full text of the 40 included abstracts by the first two authors. Criteria for inclusion upon full-text review required articles to report hazard or odds ratios for associations between AGR and any prognostic variable in a population with any form of cancer of the GI tract. After applying the full-text criteria, 13 studies were identified to meet all standards for inclusion within the systematic review (Figure 1).

Demographic information for all 13 studies, including the date published, population size, cancer analyzed, prognostic variable assessed, and hazard or odds ratios with associated 95% confidence intervals and multivariate p-values, were tabulated (Table 2).

**Table 2:** Demographic information and extracted data from included studies. This table reports demographic results from all included studies and associated HR with CI and multivariate p-values. PY: publication year; OS: overall survival; DFS: disease-free survival; RFS: recurrence-free survival; ACM: all-cause mortality; PFS: progression-free survival; RCSS: rectal cancer-specific survival; OR: odds ratio.

Author, PY	Cancer analyzed	Prognostic variable reduced	Population size	Cut-off value	Hazard ratio	Confidence interval	Multivariate P-value
Atsumi et al. (2021) [11]	Esophageal	OS	105	<1.48	2.39	1.26 - 4.55	0.008
Zhang et al. (2023) [13]	Esophageal	OS	571	<1.43	1.56	1.08 - 2.26	0.018
Zhang et al. (2016) [14]	Esophageal	OS	458	<1.30	1.32	0.88 - 1.98	0.181
Zhang et al. (2016) [14]	Esophageal	DFS	458	<1.30	1.37	0.93 - 2.09	0.114
Atsumi et al. (2021) [11]	Esophageal	RFS	105	<1.48	1.72	1.00 - 2.96	0.049
Mao et al. (2017) [10]	Gastric	OS	862	<1.50	1.71	1.03 -2.85	0.039
Xiao et al. (2019) [15]	Gastric	OS	3266	<1.80	1.13	1.00 - 1.28	0.048
Xue et al. (2017) [16]	Gastric	OS	269	<1.36	1.470 (OR)	1.02 - 2.13	0.041
Liu et al. (2017) [17]	Gastric	ACM	507	<1.93	1.489	1.00 - 2.21	0.048
Fujikawa et al. (2017) [9]	Colon	OS	248	<1.32	2.67	1.33 - 5.36	0.0058
Hu et al. (2020) [18]	Colon	OS	180	<1.33	4.35	2.07 - 9.12	0.0001
Fujikawa et al. (2017) [9]	Colon	DFS	248	<1.32	2.93	1.34 - 6.69	0.0072
Shibutani et al. (2015) [19]	Colorectal	OS	66	<1.25	2.25	1.07 - 4.73	0.033
Cho et al. (2023) [20]	Colorectal	OS	1378	<1.67	2.38	0.44 - 12.87	0.314
Shibutani et al. (2015) [19]	Colorectal	PFS	66	<1.25	2.662	1.09 - 6.53	0.033
Hu et al. (2020) [18]	Rectal	OS	181	<1.33	N/A	N/A	0.366
Li et al. (2015) [21]	Rectal	RCSS	293	<1.20	1.008	0.37 - 2.73	0.988

All studies that reported a hazard ratio (HR) analyzing the same prognostic variable in a common cancer population were subjected to generic inverse variance and random effect meta-analysis. Meta-analysis was performed in ReviewManager 5.4 (Cochrane Collaboration, London, UK), along with generated forest plots. The I^2^ test was used to assess for heterogeneity between analyzed studies. Pooled adjusted HRs with 95% confidence intervals and p-values were reported.

Results

**Figure 2:**
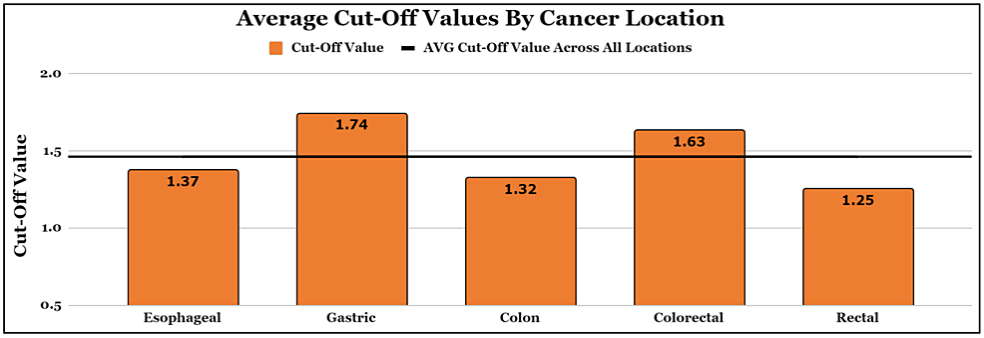
Average cut-off values for overall survival by cancer location. This figure depicts the average cut-off values by cancer location weighted by population size.

A total of 8,384 patients across 13 independent studies were included in this review and meta-analysis (Figure 1). In total, five different cancer populations based on location were analyzed: esophageal, gastric, colon, colorectal, and rectal (Table 2). The weighted average cut-off value across all populations was 1.46 (Figure 2).

Meta-analysis for the association between a low AGR and below-average overall survival (OS) was conducted for five independent outcomes: primary GI tract cancer, esophageal cancer, gastric cancer, colon cancer, and colorectal cancer (Table 3). Analysis of all primary GI cancers at a 95% confidence interval revealed a pooled adjusted HR of 1.82 (p < 0.001) (Table 3).

**Table 3:** Meta-analysis results. This table includes the meta-analysis results of reduced AGR in various outcomes based on a 95% confidence interval (CI). OS: overall survival; AGR: albumin-to-globulin ratio.

Outcome assessed	Pooled adjusted HR	95% CI	P-value	I^2^ test
Primary GI tract cancer OS	1.82	1.35 - 2.45	<0.001	71%
Esophageal cancer OS	1.57	1.19 - 2.06	0.001	14%
Gastric cancer OS	1.29	0.88 - 1.88	0.19	58%
Colon cancer OS	3.36	2.02 - 5.58	<0.001	0%
Colorectal cancer OS	2.27	1.15 - 4.48	0.02	0%

**Figure 3:**
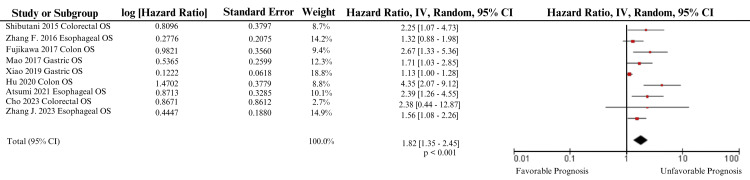
Overall survival across all primary gastrointestinal cancers - meta-analysis. This figure depicts the forest plot generated for the association between overall survival and any primary gastrointestinal cancer based on a 95% confidence interval [9-11,13-15,18-20].

The meta-analysis forest plot for the association between a low AGR and below-average OS is included in Figure 3. The percentage weight each study was allocated in a meta-analysis based on the precision of the reported hazard ratio is listed in Figure 3. The study that carried the highest meta-analysis weight was Xiao et al. (2019), which reported an HR of 1.13 (1.00-1.28), while the study that carried the least meta-analysis weight was Cho et al., which reported an HR of 2.38 (0.44-12.87) (Table 2 and Figure 3) [15,20]. The I^2^ test to screen for heterogeneity revealed an I^2^ of 71% for this calculation (Table 3).

**Figure 4:**
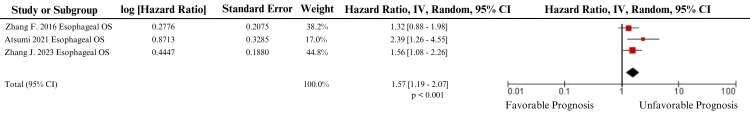
Overall survival in esophageal cancer - meta-analysis. This figure depicts the forest plot generated for the association between overall survival and esophageal cancer based on a 95% CI [11,13,14].

In esophageal cancer, a total of 1,697 patients across five independent studies were included in this review and meta-analysis (Table 2). A low AGR was assessed for an association with below-average outcomes in three prognostic variables: OS, disease-free survival (DFS), and recurrence-free survival (RFS) (Table 2). The weighted average AGR cut-off value for esophageal cancer was 1.37 (Figure 2). Meta-analysis for the association between a low AGR and below-average OS in esophageal cancer at a 95% CI revealed a pooled adjusted HR of 1.57 (p = 0.001) (Table 3). This indicates that patients with an AGR value below 1.37 were 1.57 times more likely to experience reduced OS on average, compared to age-matched and cancer-type-matched controls with an AGR value above 1.37. The meta-analysis forest plot for the association between a low AGR and below-average OS in esophageal cancer is included in Figure 4. The study that carried the highest meta-analysis weight was Zhang et al., which reported an HR of 1.56 (1.08-2.26), while the study that carried the least meta-analysis weight was Atsumi et al., which reported an HR of 2.38 (0.44-12.87) (Table 2 and Figure 4) [11,13]. The I^2^ test to screen for heterogeneity revealed an I^2^ of 14% for this calculation (Table 3).

**Figure 5:**
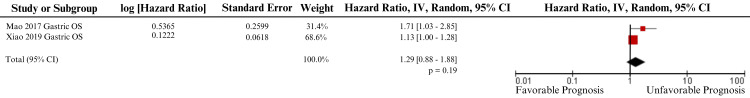
Overall survival in gastric cancer - meta-analysis. This figure depicts the forest plot generated for the association between overall survival and gastric cancer based on a 95% CI [10,15].

In gastric cancer, a total of 4,904 patients across four independent studies were included in this review and meta-analysis (Table 2). A low AGR was assessed for an association with below-average outcomes in two prognostic variables: OS and all-cause mortality (ACM) (Table 2). The weighted average AGR cut-off value for gastric cancer was 1.74 (Figure 2). Meta-analysis for the association between a low AGR and below-average OS in gastric cancer at a 95% confidence interval revealed a pooled adjusted HR of 1.29 (p = 0.19) (Table 3). While included studies found slight significance in associations for low AGR and reduced OS, a meta-analysis in our study did not find significance. The meta-analysis forest plot for the association between a low AGR and below-average OS in gastric cancer is included in Figure 5. The study that carried the highest meta-analysis weight was Xiao et al., which reported an HR of 1.13 (1.00-1.28), while the study that carried the least meta-analysis weight was Mao et al. (HR = 1.71, 1.03-2.85) (Table 2 and Figure 5) [10,15]. The I^2^ test to screen for heterogeneity revealed an I^2^ of 58% for this calculation (Table 3).

**Figure 6:**
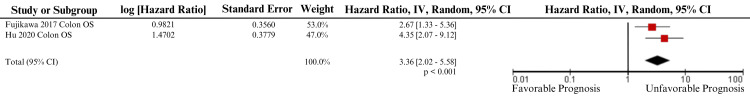
Overall survival in colon cancer - meta-analysis. This figure depicts the forest plot generated for the association between overall survival and colon cancer based on a 95% CI [9,18].

In colon cancer, a total of 676 patients across three independent studies were included in this review and meta-analysis (Table 2). A low AGR was assessed for an association with below-average outcomes in two prognostic variables: OS and DFS (Table 2). The weighted average AGR cut-off value for colon cancer was 1.32 (Figure 2). Meta-analysis for the association between a low AGR and below-average OS in colon cancer at a 95% confidence interval revealed a pooled adjusted HR of 3.36 (p < 0.001) (Table 3). This indicates that patients with an AGR value below 1.32 were 3.36 times more likely to experience reduced OS on average, compared to age-matched and cancer-type-matched controls with an AGR value above 1.32. The meta-analysis forest plot for the association between a low AGR and below-average OS in colon cancer is included in Figure 6. The study that carried the highest meta-analysis weight was Fujikawa et al., which reported an HR of 2.67 (1.33-5.36), while the study that carried the least meta-analysis weight was Hu et al. (HR = 4.35, 2.07-9.12) (Table 2 and Figure 6) [10,15]. The I^2^ test to screen for heterogeneity revealed an I^2^ of 0% for this calculation (Table 3).

**Figure 7:**
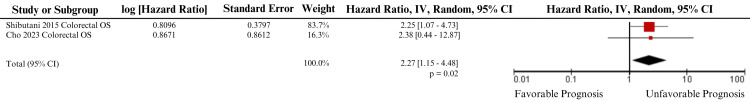
Overall survival in colorectal cancer - meta-analysis. This figure depicts the forest plot generated for the association between overall survival and colorectal cancer based on a 95% CI [19,20].

In colorectal cancer, a total of 1510 patients across three independent studies were included in this review and meta-analysis (Table 2). A low AGR was assessed for an association with below-average outcomes in two prognostic variables: OS and progression-free survival (PFS) (Table 2). The weighted average AGR cut-off value for colorectal cancer was 1.63 (Figure 2). Meta-analysis for the association between a low AGR and below-average OS in colorectal cancer at a 95% confidence interval revealed a pooled adjusted HR of 2.27 (p = 0.02) (Table 3). This indicates that patients with an AGR value below 1.63 were 2.27 times more likely to experience reduced OS on average, compared to age-matched and cancer-type-matched controls with an AGR value above 1.63. The meta-analysis forest plot for the association between a low AGR and below-average OS in colorectal cancer is included in Figure 7. The study that carried the highest meta-analysis weight was Shibutani et al., which reported an HR of 2.25 (1.07-4.73), while the study that carried the least meta-analysis weight was Cho et al. (HR = 2.38, 0.44-12.87) (Table 2 and Figure 7) [19,20]. The I^2^ test to screen for heterogeneity revealed an I^2^ of 0% for this calculation (Table 3).

In rectal cancer, a total of 474 patients across two independent studies were included in this review and meta-analysis (Table 2). A low AGR was assessed for an association with below-average outcomes in two prognostic variables: OS and rectal cancer-specific survival (RCSS) (Table 2). The weighted average AGR cut-off value for rectal cancer was 1.25 (Figure 2). A meta-analysis was not performed for the association between a low AGR and reduced OS in rectal cancer because only one included study analyzed OS. Both included rectal cancer studies did not find significance in their experiments (Table 2).

**Figure 8:**
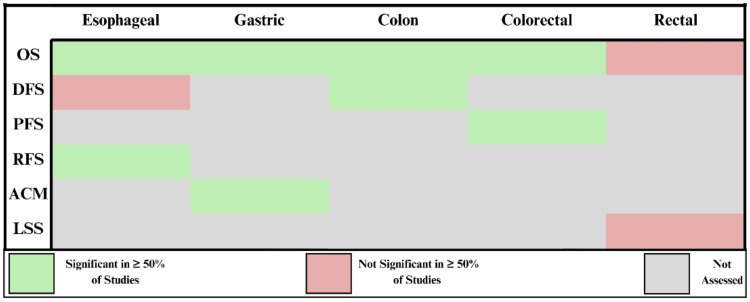
Summary of all AGR prognostic analyses identified and current gaps in the literature. This figure depicts prognostic variables assessed for significance. Variables assessed were reported as either significant or not significant in the majority of studies. AGR: albumin-to-globulin ratio; OS: overall survival; DFS: disease-free survival; RFS: recurrence-free survival; ACM: all-cause mortality; PFS: progression-free survival; LSS: location-specific survival.

All cancer-prognostic variable combinations extracted from the literature were reported in Figure 8. OS was the most common prognostic variable extracted, being analyzed in all five populations (Figure 8). Conversely, PFS and location-specific survival (LSS) were the least common prognostic variables, being analyzed in only one population each (Figure 8).

Discussion

The goal of this systematic review and meta-analysis was to compile all available research reporting the use of AGR as a prognostic indicator for primary GI malignancies to determine which malignancies show AGR to be a statistically significant prognostic indicator. AGR is a standard component of serum metabolic panels in routine medical practice, making it a readily available, inexpensive, and efficient tool to contribute to the clinical determination of prognosis for physicians as they care for their cancer patients.

Interestingly, the AGR cut-off for OS in esophageal cancer was 1.37, which is below the cut-off value for AGR for GI cancers as a whole for those analyzed in this study (Figure 2). A high AGR cut-off value indicates a higher threshold of the patient's overall nutrition and immune status to be considered to have a favorable prognosis. This is slightly paradoxical given the very aggressive nature of esophageal cancer as it is the sixth leading cause of cancer-related death and holds a five-year survival rate of 15-25% [22]. This low AGR cut-off value for esophageal cancer patients creates a low threshold for clinicians to operate and be aggressive with treatment options to expand life expectancy.

Similarly, the AGR cut-off values for OS in the gastric cancer and colorectal cancer groups were the two highest cut-off points (Figure 2). The liver is the site of synthesis for albumin and the majority of the components that make up serum globulin [23]. The liver receives a large majority of its blood supply from the hepatic portal vein, thus the liver is very sensitive to imbalance within the GI tract [24]. In response to pro-inflammatory cytokines associated with the inflammatory state, the liver responds by producing acute-phase reactants and suppression of albumin synthesis [14]. Thus, in the setting of hypoalbuminemia and hyperglobulinemia, the potentiated inflammatory response fosters an optimal environment for cancer development and proliferation [25]. This is consistent with the increased AGR cut-off values as a higher threshold is needed for a patient to have a favorable prognosis.

Interestingly, AGR was not found to be significantly associated with OS in gastric cancer upon meta-analysis, despite being significant in both included studies that reported it (Tables 2, 3). The research team has a few potential hypotheses for this finding. First, the stomach is different from all other portions of the alimentary canal analyzed by this review in that its luminal volume is much larger. Thus, gastric cancer is less likely to cause a mechanical obstruction than the portions of the GI tract with smaller lumens. This reduced anatomic effect of gastric cancer may significantly decrease the cancer’s mechanical effect on downstream nutrient absorption when compared to other GI cancers. Secondly, a significant portion of the stomach's immune defense is stomach acid. While stomach acid is effective in defending against many microorganisms, it possesses no efficacy in preventing cancer. For this reason, globulin levels may not be as sensitive to gastric cancer as they are to other GI cancers. Lastly, only two studies were included that assessed AGR in OS for gastric cancer, indicating the need for more research to explore this relationship further.

There are some limitations to this systematic review and meta-analysis that should be addressed. In an effort to produce the most accurate results possible, the inclusion criteria for this review were highly specific. Additionally, the majority of studies included in this review were completed in continental Asia. The impact of different cultural diets on albumin levels has not been precisely defined. As mentioned in this study, nutritional status possesses a strong influence on albumin levels. The variation between different cultural diets may impact the albumin levels and subsequently AGRs. This potential influence may impact the generalizability of our study to other geographic regions. Future research analyzing the impacts of specific cultural diets on albumin levels would likely prove beneficial.

## Conclusions

Overall, AGR is shown to be a valuable prognostic indicator in various types of GI-related malignancies. It is because of this correlation combined with its accessibility, ease of use, and affordable cost profile that AGR should always be used in prognostic estimation as well as an additional tool in determining the appropriate treatment for each individual patient with a GI cancer diagnosis. To date, research into the clinical use of AGR has only focused on a small subset of variables. Expanding the scope of research into AGR's potential correlation with more prognostic variables can open a door to combating the threat that GI malignancies pose to society. The clinical use of AGR to accurately deliver prognosis and guide clinical decision-making will undoubtedly improve patient care.
